# Coronary Artery Disease: Association Study of 5 Loci with Angiographic Indices of Disease Severity

**DOI:** 10.1155/2021/5522539

**Published:** 2021-07-12

**Authors:** Neda M. Bogari, Reem M. Allam, Abdellatif Bouazzaoui, Osama Elkhateeb, Massimo Porqueddu, Gualtiero I. Colombo

**Affiliations:** ^1^Department of Medical Genetics, Faculty of Medicine, Umm Al-Qura University, Makkah, KSA, Saudi Arabia; ^2^Department of Clinical Pathology, Faculty of Medicine, Zagazig University, Egypt; ^3^Science and Technology Unit, Umm Al-Qura University, Makkah, Saudi Arabia; ^4^Department of Cardiology, King Abdullah Medical City Makkah, Makkah, Saudi Arabia; ^5^Department of Cardiology, Dalhousie University Halifax, Nova Scotia, Canada; ^6^Department of Cardiac Surgery, King Fahd Armed Forces Hospital, Jeddah, Saudi Arabia; ^7^Department of Cardiac Surgery, Centro Cardiologico, Monzino, Milan, Italy; ^8^Unit of Immunology and Functional Genomics, Centro Cardiologico Monzino IRCCS, Milan, Italy

## Abstract

**Background:**

Different common gene variants were related to coronary artery disease (CAD) in many studies. Yet, the relation of these loci to the severity of CAD is not completely elucidated.

**Methods:**

We enrolled 520 subjects (315 CAD cases and 205 controls). CAD presence and extension were assessed by coronary angiography (CAG). Genotyping of five SNPs (namely, rs2230806 (1051G > A) in ABCA1 on chromosome 9, rs2075291 (553G > T) in ApoA5 on chromosome 11, rs320 in LPL on chromosome 8 intron (T → G at position 481), rs10757278 (c.22114477A > G), and rs2383206 (c.22115026 A > G) on chromosome 9p21 locus) was performed by allele-specific PCR. The degree and site of arterial lesions were used to classify patients, tested for association with CAD severity, and related to allele dosage.

**Results:**

The polymorphisms rs2383206 and rs10757278 showed significant associations with 2- and 3-vessel coronary disease (p =0.003 and 0.006, respectively). The homozygous GG genotypes of rs10757278 was associated with higher frequency of left anterior descending (LAD), right coronary artery (RCA) and left circumflex (LCX) diseases (p =0.002, 0.016 and 0.002, respectively). The GG genotypes of rs2383206 were found in higher percentage in patients with left main (LM) trunk and left circumflex (LCX) diseases (*p* = 0.013 and 0.002, respectively).

**Conclusion:**

SNPs rs10757278 and rs2383206 allele dosage could predict CAD severity in the Saudi Arab population.

## 1. Introduction

Despite major advances in patient management, coronary artery disease (CAD) remains a leading cause of death worldwide [1]. CAD underlies a composite group of syndromes caused by partial or complete cardiac muscle regional ischemia as a result of temporary or permanent cessation of blood flow in one or more of coronary arteries [2]. These can be diagnosed by clinical, laboratory, and angiographic assessment of the patients suffering from chest pain [3].

Coronary angiography is a technique that uses contrast dye, containing iodine, and X-ray images to detect coronary artery stenosis that is caused by atheromatous plaque development and/or rupture [4, 5]. The plaque pathogenesis is a complex, multistep, and multietiological process [6]. Genetic bases of this process have been recently studied in a wide series of genome-wide association studies [7–11].

Former studies proved that different genetic variants were associated with the major traditional risk factors of CAD [12–15]. Interestingly, genetic factors have been shown to increase the risk of early-onset CAD [12, 16, 17]. Therefore, elucidating the molecular mechanisms and genetic predisposition to the disease could permit early detection of individuals at higher risk for CAD, and this, in turn, could pave the way to targeted preventive therapies [18–20]. Several genetic loci on chromosome 9p21.3 and other chromosomes are claimed to have a prominent role in CAD development [21–24].

To the best of our knowledge, few works in the literature studied the relation between genetic risk of CAD and severity indices of the disease [25, 26]. Our objective is to spot the light on angiographic indices of CAD severity in relation to relevant genetic variants in the Saudi Arab population. Five SNPs at 3 loci were selected according to data from previous literature, as they had shown a strong relation with CAD risk [27–36]. We investigated their relation to the disease severity.

## 2. Materials and Methods

### 2.1. Study Design and Subjects

Our study is a prospective case-control study on unrelated Arab individuals in Saudi Arabia. Three hundred and fifteen consecutive CAD patients between 30 and 85 years of age were enrolled from the Noor Specialty Hospital (NSH) and King Abdullah Medical City (KAMC) of Mecca. All patients underwent coronary angiography on admission. The number of coronary vessels affected and the degree of atherosclerotic lesions were assessed. CAD patients were classified according to their angiographic data into 2 groups, group I (nonobstructive CAD) with nonsignificant lesions, i.e., stenosis < 50%, consists of 32 patients, and group II (obstructive CAD) consists of 283 patients, i.e., with stenosis ≥ 50% (significant lesions) in at least one main coronary artery. Group II (obstructive CAD) was further subdivided into 3 subgroups according to the number of vessels affected.

Population-based control individuals (*n* = 205) were enrolled in the study after filling a questionnaire upon voluntary blood donation. A detailed medical history and a complete clinical examination were performed, along with vital signs and anthropometric measurements, to rule out cardiac problems.

We exclude patients with autoimmune diseases, cancer, and other comorbidities that may affect variables under study.

The body mass index (BMI) cut-points were as specified by the World Health Organization (WHO) criteria. It was computed by dividing the weight (kg) by square height (m^2^) [37]. History of hypertension (HTN) was confirmed by assessing systolic (SBP) and diastolic blood pressure (DBP): an SBP of ≥140 and/or a DPB ≥ 90 mmHg was considered indicative of HTN. Type 2 diabetes mellitus (T2DM) was defined according to WHO criteria [38]. Dyslipidemia was diagnosed according to the National Cholesterol Education Program Adult Treatment Panel III criteria [39].

The procedure of the study was authorized by the Institutional Review Boards of the Umm Al-Qura University (UQU) (43430838) and KAMC (IRB number 13-043). Written consent was obtained from each participant.

### 2.2. Angiography

The coronary angiograms were reviewed blindly to genotype results by 2 independent cardiologists. Stenosis in any vessel ≥ 50% was considered as significant. Left main disease was defined as stenosis ≥ 50% in a nonbypassed vessel. Protected left main disease was excluded from analysis, as causing enhanced atherosclerosis in the segments proximal to a bypass anastomosis. [40]

### 2.3. Biochemical Assays

We collected two tubes of peripheral blood from each subject, an EDTA-tube for DNA extraction and a plain tube for glucose and lipids biochemical assay. The biochemical tests were performed at KAMC clinical chemistry lab. The genetic study was done at Umm Al-Qura University biomedical labs.

### 2.4. Genotyping

Five SNPs at 3 loci were studied: rs320 in lipoprotein lipase (LPL) on chromosome 8 intron (T → G at position 481); rs2230806 (1051G > A) in ABCA1, rs10757278 (c.22114477A > G), and rs2383206 (c.22115026 A > G) on chromosome 9; and rs2075291 (553G > T) in ApoA5 on chromosome 11.

Following the manufacturer's protocol, DNA extraction was performed from whole blood samples of all subjects, using the EZ1 DNA Blood 350 *μ*l Kit (Qiagen, Hilden, Germany). A NanoDrop spectrophotometer was used to assess DNA concentration and purity. Allele-specific PCR amplification was used for genotyping. Primer design allowed specific amplification of the reference and minor alleles. TaqMan MGB probe and allele-specific primers were provided on demand (Applied Biosystems, Foster City, CA). We performed the 5′ nuclease assay by using 20 ng of genomic DNA, 1× primer/probe mix, and 1× TaqMan Universal PCR Master Mix (Applied Biosystems). The cycling conditions needed for amplification were performed according to the manufacturer's recommendations.

### 2.5. Statistical Analysis

The Kolmogorov-Smirnov normality test examines if variables are normally distributed.

Mean ± standard deviation was used to express normally distributed quantitative continuous data, while median ± interquartile range (IQR) was used to express nonnormally distributed continuous data. Frequencies and percentages were used for categorical data.

Student's *t*-test and ANOVA were used to assess differences in continuous variables among groups, as appropriate. Mann–Whitney *U* test was used to compare nonnormally distributed variables. We assessed the Hardy-Weinberg equilibrium and compared genotype distribution and allele frequencies between the study groups by 2 × 2 contingency tables and *χ*^2^ test. Logistic regression analysis was performed and adjusted for confounders to calculate odds ratios (ORs) with 95% CI and corresponding *p* values, with minor homozygous allele as the reference group. Two-tailed *p* value < 0.05 was considered statistically significant. The SPSS program for Windows was used for statistical analysis (version 23; Texas instruments, IL, USA).

## 3. Results

### 3.1. Characteristics of the Study Population

Patients (*n* = 315) with angiographically confirmed CAD were enrolled. Male patients represent 62.2% of the cohort. The mean age of cases was 59.2 ± 10.5 years.

Characteristics of the demographic and clinical parameters of cases and controls are presented in Table 1. Risk factors were significantly more frequent and/or higher in obstructive-CAD patients when compared with either nonobstructive CAD patients or controls. Dyslipidemia and hypertension frequencies were lower in controls, but more similar between nonobstructive and obstructive CAD patients.

Patients with 1-vessel, 2-vessel, and 3-vessel disease were 55 (19.5%), 104 (36.7%), and 124 (43.8%), respectively. The percent of patients with left main (LM) disease was 8.7%, with left anterior descending (LAD) lesions 76.8%, with right coronary artery (RCA) lesions 67.2%, and with left circumflex (LCX) lesions 51.6%.

### 3.2. Genotype Frequencies

**Figure 1:**
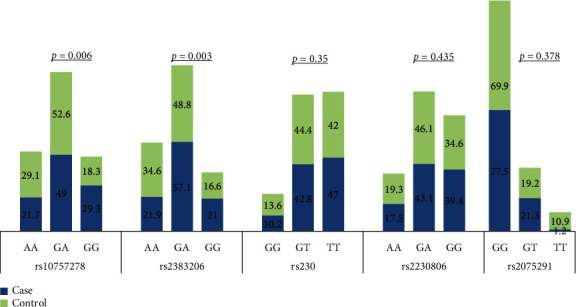
Genotype distribution among CAD patients and controls.

In the population studied, there was no significant deviation from Hardy-Weinberg Equilibrium (*p* > 0.05) for all examined polymorphisms. We observed a significant difference in the frequency distribution of the genotypes of rs10757278 and rs2383206 among cases and controls (*p* = 0.006 and *p* = 0.003, respectively), as shown in Figure 1. Conversely, genotype distribution was not significantly different for rs320, rs2230806, and rs2075291 between cases and controls with no history of CAD (*p* = 0.353, *p* = 0.435, and *p* = 0.378, respectively). Data is available as (Supplementary Table 1).

**Figure 2:**
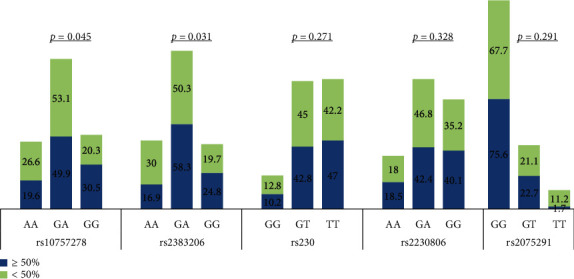
Genotype distribution among CAD patients according to the severity of angiographic stenosis.

Genotype distribution of rs10757278 and rs2383206 showed significant differences also when comparing group I and group II patients (*p* = 0.045 and 0.031, respectively). The differences in genotype distribution were not significant for the remaining SNPs (Figure 2). Data is available as (Supplementary Table 2).

### 3.3. Genetic Alleles and Risk for Obstructive Coronary Artery Disease

Genetic association of the minor alleles was assessed by calculating the OR along with CI 95% for all SNPs. The G allele of rs10757278 was found to increase the odds of CAD among cases when compared to controls by 1.409 folds (95%CI = [1.131 − 1.756]; *p* = 0.001). The risk was increased to 3.38 folds when the OR was calculated comparing CAD cases with nonobstructive CAD subjects (Table 2).

Also, the G allele of rs2383206 was found to increase the odds of CAD among cases when compared to controls by 1.413 folds (95%CI = [1.099 − 1.817]; *p* = 0.004). The risk was increased to 2.925 folds when the OR was calculated comparing obstructive vs. nonobstructive CAD (Table 2).

The other SNPs investigated did not reveal any significant association between genetic variants with CAD risk among the study subjects.

### 3.4. Risk Assessment for the Severity of CAD Lesion

Binary logistic regression test comparing subjects with significant lesions vs. nonsignificant ones detected several independent variables that increase the risk of stenosis. After adjusting confounding factors, regression analysis showed that smoking (OR = 2.81, *p* = 0.032), diabetes (OR = 1.92, *p* = 0.041), and the G allele of rs10757278 and rs2383206 (OR = 1.82 and 2.41, *p* = 0.015 and 0.036, respectively) increased the risk of coronary stenosis independently of the other variables (Table 3).

### 3.5. Association between Genotypes and the Number and Location of Coronary Lesions

A strong relation between the dosage of the G allele of rs10757278 and the number of diseased vessels in obstructive CAD patients (Table 4). Results showed that individuals with rs10757278 GG genotype had a significantly higher chance of getting 2- or 3-vessel disease (*p* ≤ 0.001; OR = 1.952, 95% CI: 1.131–3.348 and *p* = 0.006; OR = 2.854, 95% CI: 1.264–6.192, respectively). Conversely, no significant association was found between rs10757278 G allele dosage and 1-vessel disease. Higher rates of LAD stenosis (*p* = 0.001, OR = 2.284, 95%CI = 1.271–3.521), RCA stenosis (*p* = 0.004, OR = 1.261, 95%CI = 1.241–3.318), and LCX stenosis (*p* = 0.002, OR = 1.121, 95%CI = 1.041–2.261) were found in GG genotype subjects (Table 4).

Similar strong associations were found for the GG genotype of rs2383206 with CAD patients with two or three-vessel disease (*p* = 0.004, OR = 1.723, 95% CI: 1.328-3.452 and *p* = 0.003, OR = 1.862, 95% CI: 1.175-4.153, respectively), but not with 1-vessel disease (Supplementary Table S3). The GG genotype is found to be associated more with LM stenosis (*p* = 0.013, OR = 2.382, 95%CI = 1.061–4.812) and LCX stenosis (*p* = 0.002, OR = 2.512, 95%CI = 1.437–4.038) (Supplementary Table S3).

## 4. Discussion

In our study, CAD cases and controls were precisely stratified according to the angiographic estimation of the number and/or degree of stenosis of coronary artery lesions. The severity of CAD was assessed angiographically as the number and degree of coronary artery stenosis. Association between genetic variants and different risk factors was assessed by careful clinical and biochemical characterization of study subjects. Our results showed a significant association between risk allele frequency of SNPs rs10757278 and rs2383206 and CAD angiographic severity.

Our findings are consistent with different GWAS that proved an association of 9p21 locus polymorphism and CAD in different ethnic groups [7, 41–45]. McPherson et al. described two SNPs (rs10757274 and rs2383206) on the 9p21 chromosome that were found to be in association with CAD among Caucasians [43]. Helgadottir et al. showed that almost 21% of subjects of European ancestry carry a homozygous genotype for rs10757278-G and assessed a risk of 1.64-fold higher than the noncarriers to have MI [42]. Shen et al. considered 4 SNPs in Korean population on chromosome 9p21, which were found related to CAD (rs2383206 and rs10757274) and MI (rs10757278 and rs2383207). They defined CAD severity as ≥70% coronary stenosis and characterized a greater cross-race risk for CAD development [46].

A collaborative meta-analysis, including 21 studies, investigated the association between the chromosome 9p21 locus and the burden of angiographic CAD [47]. Risk allele homozygotes are 23% more prone to multivessel disease than to single-vessel disease when compared with nonrisk allele homozygotes. Our results in a Saudi Arab cohort concerning 2 SNPs on chromosome 9p21 locus go in parallel with their findings.

For the ABCA1 R219K variant (+1051GA, rs2230806), we did not find any significant difference in genotype distribution or association with disease risk and/or severity between patients and controls. This is in line with Takagi et al. who concluded that the polymorphism does not seem to influence coronary atherosclerosis [48]. On the contrary, a meta-analysis including 2658 controls and 2730 CAD patients found that the ABCA1 gene K allele had a significant role in protecting against the risk of CAD in Chinese and was associated with decreased CAD susceptibility [49]. Indeed, several studies highlighted a protective effect of the rs2230806 polymorphism [50, 51]. On the opposite side, Brousseau et al. found that rs2230806 could potentiate the development of CAD [52]. Abd El-Aziz et al. found that the ABCA1 gene R allele was associated with premature CAD in the Egyptian population: the SNP had an apparent impact on patients with low HDLc [53].

The SNP rs2075291 (553G > T) is a rare APOA5 gene variant. Our results matched a large meta-analysis that included 3 studies with 9,518 subjects, which found no association between rs2075291 (553G > T) gene polymorphism and risk of CHD [54]. Besides, the SNP rs2075291 was found to be correlated with triglycerides and total cholesterol serum levels in Chinese Han population [55]. An association between rs2075291 polymorphism and CHD was found to be significant in Taiwan and Nanjing cohorts [56, 57].

We found no difference in LPL polymorphisms genotype distribution between the control and CAD group. Thus, rs320 could not be considered as independent CAD risk factors in our population. These results go in parallel with others in different regions in the Kingdom of Saudi Arabia [33, 35, 36], but do not replicate the results Al-Jafari et al. who found a significant association between this polymorphism and the risk of CAD [34]. An Indian study strongly suggested that the homozygous genotype of rs320 on the LPL gene is an independent risk factor for first MI [58].

In conclusion, our study confirmed that the presence of the G allele in rs10757278 and rs2383206 was significantly associated with multivessel CAD, while rs2230806, rs2075291, and rs320 did not significantly affect CAD.

To our knowledge, this is the first study to investigate the association of 5 CAD-associated SNPs with the occurrence, extent, and severity of the disease. We had to acknowledge some limitations of our research. Linkage study could not be performed among the 5 loci under study due to their positions on different chromosomes. Increasing the number of SNPs in the same chromosome in further research could help to perform linkage analysis. Another limitation is that we did not classify CAD patients clinically. Other research encompassed several clinical CAD presentations including MI, calcification in coronary arteries, or angiographic CAD plus MI history. Further prospective cohort studies with larger sample sizes are needed to confirm our results and evaluate the prognosis and degree of atherosclerosis progression.

## Figures and Tables

**Table 1:** Demographics and clinical characteristics of study groups.

	Control (*n* = 205)	Nonobstructive CAD (*n* = 32)	Obstructive CAD (*n* = 283)
A. Demographics			
Age (years)	58.4 ± 9.28	58.7 ± 10.4	59.3 ± 10.5
Male (*n* (%))	119 (58%)	27 (90%)	244 (60.1%)^†^
B. Clinical parameters			
Body mass index (kg/m^2^)	26.26 (6.49)	28.2 ± 4.0^§^	28.5 ± 5.0^§^
Systolic blood pressure (mmHg)	128.2 ± 5.8	140.5 ± 9.4^§^	143.1 ± 9.9^§^
Diastolic blood pressure (mmHg)	79.3 ± 2.8	83.3 ± 3.7^§^	85.0 ± 4.0^§^
Previous stroke (%)	7 (3.4%)	4 (13.3%)^∗^	75 (18.5%)^§^
Diabetes mellitus (%)	35 (17.1%)	13 (43.3%)^§^	198 (48.8%)^§^
Current smokers (%)	72 (35.1%)	27 (90%)^§^	164 (40.4%)^‡^
Exercise (%)	67 (32.7%)	10 (33.3%)^§^	104 (25.6%)^‡^
Fasting blood sugar (mg/dl)	138.3 ± 6.2	146.6 ± 8.6^∗^	163.9 ± 8.1^§^
Total cholesterol (mg/dl)^#^	177.9 ± 69.9	241.4 ± 4.7^§^	242.3 ± 7.5^§^
HDL-C (mg/dl)	38.7 ± 10.4	39.1 ± 2.0^§^	38.7 ± 2.4^§^
LDL-C (mg/dl)	91.9 ± 3.9	174.2 ± 4.3^§^	185.2 ± 4.9^§^
Triglyceride (mg/dl)^#^	191.2 ± 4.1	225.2 ± 5.2^§^	237.9 ± 6.9^†§^

Student *t* test and *X*^2^ test between obstructive CAD and either control groups. ^#^Mann–Whitney *U* test vs. control group: ^∗^*p* < 0.01, ^§^*p* < 0.001 vs. subjects with nonobstructive CAD: ^‡^*p* < 0.01, ^†^*p* < 0.001.

**Table 2:** Risk alleles in CAD subjects compared with control groups.

SNP variant	Risk allele OR (95% CI) Obstructive CAD/control	*p*	Risk allele OR (95% CI) Obstructive/nonobstructive CAD	*p*
rs10757278	1.409 (1.131–1.756)	0.001	3.38 (1.231–6.341)	0.006
rs2383206	1.413 (1.009–1.718)	0.004	2.925 (1.132–4.562)	0.012
rs320	0.826 (0.635–1.074)	0.087	1.262 (0.973–2.326)	0.151
rs2230806	1.253 (0.761–1.941)	0.091	1.430 (0.901–2.182)	0.163
rs2075291	0.564 (0.327–0.132)	0.128	0.705 (0.561–0.182)	0.231

**Table 3:** Risk factors contributing to the severity of CAD.

Variable	B	SE	*p*	OR	CI
rs10757278 G	0.273	0.117	0.015	1.82	(1.17-2.57)
rs2383206 G	0.332	0.142	0.003	2.41	(1.31-3.53)
Smoking (current + ever)	0.782	0.139	0.032	2.81	(1.52-4.03)
DM	0.853	0.171	0.041	1.92	(1.23-2.71)

CAD: coronary artery disease; DM: diabetes mellitus; SE: standard error; CI: confidence interval.

**Table 4:** Association between SNP rs10757278 genotypes and severity of CAD.

		Reference genotype	AG			GG		
			OR	95% CI	*p*	OR	95% CI	*p*
Number of lesions	1VD	AA	0.720	0.365-1.397	0.401	1.207	0.635-2.243	0.260
	2VD	AA	0.787	0.351-1.533	0.541	1.952	1.131-3.348	≤0.001
	3VD	AA	1.207	0.591-3.421	0.129	2.854	1.264-6.192	0.006
Location of lesions	LM stenosis	AA	2.254	0.510-9.964	0.265	3.478	0.674-11.262	0.115
	LAD stenosis	AA	1.109	0.619-1.701	0.691	2.284	1.271-3.521	0.001
	RCA stenosis	AA	1.044	0.505-1.651	0.845	1.261	1.241-3.318	0.004
	LCX stenosis	AA	1.119	0.581-1.705	0.677	1.121	1.041 – 2.261	0.002

SNP: single nucleotide polymorphism; CAD: coronary artery diseases; VD: vessel disease; LM: left main; LAD: left anterior descending; RCA: right coronary artery; LCX: left circumflex.

## Data Availability

The data used to support the findings of this study are available from the corresponding author upon request.

## Supplementary Materials

Supplementary Materials Tables describing genotype distribution among CAD patients and controls, genotype distribution among CAD patients according to the severity of angiographic stenosis, and association between SNP rs2383206 genotypes and CAD severity are included.
