# Supplementary material for: Coronary Artery Disease: Association Study of 5 Loci with Angiographic Indices of Disease Severity
Source: Dis Markers. 2021 Jul 12;2021:5522539. doi: 10.1155/2021/5522539 (PMC8292076; doi:10.1155/2021/5522539)
Supplement: Supplementary Materials — Tables describing genotype distribution among CAD patients and controls, genotype distribution among CAD patients according to the severity of angiographic stenosis, and association between SNP rs2383206 genotypes and CAD severity are included. [file 5522539.f1.docx]

**(Suppl S1):** Genotype distribution among CAD patients and controls

|  | **Rs10757278*** | | **Rs2383206*** | **Rs2230806** | **Rs320** | | **Rs2075291** |
| --- | --- | --- | --- | --- | --- | --- | --- |
| **Case** | **AA** | 21.7 | 21.9 | 17.5 | **GG** | 10.2 | 77.5 |
|  | **GA** | 49 | 57.1 | 43.1 | **GT** | 42.8 | 21.3 |
|  | **GG** | 29.3 | 21 | 39.4 | **TT** | 47 | 1.2 |
| **Control** | **AA** | 29.1 | 34.6 | 19.3 | **GG** | 13.6 | 69.9 |
|  | **GA** | 52.6 | 48.8 | 46.1 | **GT** | 44.4 | 19.2 |
|  | **GG** | 18.3 | 16.6 | 34.6 | **TT** | 42 | 10.9 |

⁎p<.01 by X2 test

**(Suppl S2):** Genotype distribution among CAD patients according to the severity of angiographic stenosis

|  | **Rs10757278*** | | **Rs2383206*** | **Rs2230806** | **Rs320** | | **Rs2075291** |
| --- | --- | --- | --- | --- | --- | --- | --- |
| **Non-obstructive CAD** | **AA** | 26.6 | 30 | 18 | **GG** | 12.8 | 67.7 |
|  | **GA** | 53.1 | 50.3 | 46.8 | **GT** | 45 | 21.1 |
|  | **GG** | 20.3 | 19.7 | 35.2 | **TT** | 42.2 | 11.2 |
| **Obstructive CAD** | **AA** | 19.6 | 16.9 | 18.5 | **GG** | 10.2 | 75.6 |
|  | **GA** | 49.9 | 58.3 | 42.4 | **GT** | 42.8 | 22.7 |
|  | **GG** | 30.5 | 24.8 | 40.1 | **TT** | 47 | 1.7 |

⁎p<.01 by X2 test

**(Suppl S3):** Association between SNP rs2383206 genotypes and CAD severity.

|  |  | **Referent Genotype** | **AG** | | | **GG** | | |
| --- | --- | --- | --- | --- | --- | --- | --- | --- |
|  |  |  | **OR** | **95%CI** | ***P*** | **OR** | **95%CI** | ***P*** |
| **Number of lesions** | 1VD | AA | 0.610 | 0.238-1.298 | 0.591 | 1.437 | 0.705-2.239 | 0.327 |
|  | 2VD | AA | 0.827 | 0.461-1.673 | 0.601 | 1.723 | 1.328-3.452 | **0.004** |
|  | 3VD | AA | 1.391 | 0.281-2.451 | 0.130 | 1.862 | 1.175-4.153 | **0.003** |
| **Location of lesions** | LM stenosis | AA | 1.187 | 0.345-5.734 | 0.315 | 2.382 | 1.061-4.812 | **0.013** |
|  | LAD stenosis | AA | 1.209 | 0.712-2.235 | 0.711 | 2.181 | 0.628-3. 258 | 0.132 |
|  | RCA stenosis | AA | 2.114 | 0.703-4.041 | 0.796 | 1.346 | 0.732-3.923 | 0.216 |
|  | LCX stenosis | AA | 1.341 | 0.671-1.919 | 0.312 | 2.512 | 1.437-4.038 | **0.002** |
